# Over-Expression of *HDA710* Delays Leaf Senescence in Rice (*Oryza sativa* L.)

**DOI:** 10.3389/fbioe.2020.00471

**Published:** 2020-05-19

**Authors:** Nannan Zhao, Minghao Sheng, Jie Zhao, Xuelian Ma, Qiang Wei, Qian Song, Kang Zhang, Wenying Xu, Chuanqing Sun, Fengxia Liu, Zhen Su

**Affiliations:** ^1^College of Biological Sciences, China Agricultural University, Beijing, China; ^2^Beijing Key Laboratory of Crop, Ministry of Education (MOE) Laboratory of Crop Heterosis and Utilization, National Center for Evaluation of Agricultural Wild Plants (Rice), Beijing, China; ^3^Genetic Improvement, Department of Plant Genetics and Breeding, China Agricultural University, Beijing, China

**Keywords:** *HDA710*, InDel polymorphism, leaf senescence, co-expression network, *OsGSTU12*, rice

## Abstract

Histone deacetylases (HDACs) influence chromatin state and gene expression. Eighteen *HDAC* genes with important biological functions have been identified in rice. In this study, we surveyed the gene presence frequency of all 18 rice *HDAC* genes in 3,010 rice accessions. *HDA710/OsHDAC2* showed insertion/deletion (InDel) polymorphisms in almost 98.8% *japonica* accessions but only 1% *indica* accessions. InDel polymorphism association analysis showed that accessions with partial deletions in *HDA710* tended to display early leaf senescence. Further transgenic results confirmed that *HDA710* delayed leaf senescence in rice. The over-expression of *HDA710* delayed leaf senescence, and the knock-down of *HDA710* accelerated leaf senescence. Transcriptome analysis showed that photosynthesis and chlorophyll biosynthesis related genes were up-regulated in *HDA710* over-expression lines, while some programmed cell death and disease resistance related genes were down-regulated. Co-expression network analysis with gene expression view revealed that *HDA710* was co-expressed with multiple genes, particularly *OsGSTU12*, which was significantly up-regulated in *35S:*:*HDA710-sense* lines. InDels in the promoter region of *OsGSTU12* and in the gene region of *HDA710* occurred coincidentally among more than 90% accessions, and we identified multiple W-box motifs at the InDel position of *OsGSTU12*. Over-expression of *OsGSTU12* also delayed leaf senescence in rice. Taken together, our results suggest that both *HDA710* and *OsGSTU12* are involved in regulating the process of leaf senescence in rice.

## Introduction

Epigenetic changes can reprogram the transcriptome during various biological processes. Acetylation and deacetylation of histones has emerged as a fundamental regulatory mechanism for the control of gene expression during plant development and in response to environmental conditions (Jang et al., 2003). Acetylation is a type of post-translational modification that changes as cells age, especially in animals (Sidler et al., 2017). With increasing age and senescence, H3K14ac, H4K8ac, and H4K12ac levels in mouse increase (Huang J. C. et al., 2007), and H3K56ac levels in mouse and human decrease (Dang et al., 2009; Feser et al., 2010; O'Sullivan et al., 2010), while H3K9ac and H4K16ac show different patterns in various species (Sidler et al., 2017). Histone deacetylases (HDACs) enable tightening of the chromatin structure into heterochromatin; thus, the deacetylation of histones is associated with compacting the DNA and repressing transcription. In eukaryotes, HDACs are grouped into three families: RPD3/HDA1 (Reduced Potassium Dependence 3/Histone Deacetylase 1), SIR2 (Silent Information Regulator), and HD2 (Histone Deacetylase 2)-related protein families (Pandey et al., 2002). SIRT1, a HDAC, deacetylates and inactivates the main transcriptional regulator of genes involved in inflammation processes associated with aging (Lawrence, 2009).

Plant HDACs represent a large family encoded by multiple genes, with different subcellular locations and expression profiles revealing their functional diversity (Schmid et al., 2005). Increasing evidence shows that plant HDACs play an important role in development, including flower development (Li C. et al., 2011), seed development (Wu et al., 2000), root development (Xu et al., 2005), cell proliferation and death (Nelissen et al., 2005; Huang L. et al., 2007; Bourque et al., 2011), and respond to various abiotic and biotic stresses (Sridha and Wu, 2006; Hu et al., 2011; Luo et al., 2012). Much about the functional diversity and redundancy of different HDACs remains unknown (Hollender and Liu, 2008). The RPD3/HDA1 group HDACs are further classified into three groups, among which class I is best characterized and contains four members, namely *HDA19, HDA6, HDA7*, and *HDA9* in *Arabidopsis*. The down-regulation of *Arabidopsis AtHD1* (*HDA19*) induces various developmental defects, including leaf early senescence; HDA6 and HDA19 are involved in abscisic acid (ABA) and abiotic stress responses; HDA9 can negatively regulate salt and drought responses (Chen and Tian, 2007; Chen and Wu, 2010; Zheng et al., 2016). Therefore, the level of acetylation mediated by deacetylases is critical for the senescence process and abiotic stress response of plants.

Rice is a staple food crop with two subspecies, *japonica* and *indica*, originating from tropical or subtropical areas (Zhang et al., 2017). Much work has been done in rice (*Oryza sativa* L.) to reveal the specific mechanisms involved in acquisition, inheritance, and resetting of epigenetic information (Zhao and Zhou, 2012). Rice contains 18 HDACs, which play an important role in response to abiotic stress and vegetative growth (Jang et al., 2003; Luo et al., 2017). In rice, down-regulation of RPD3/HDA1 class I type HDACs by RNAi or amiRNA leads to multiple developmental defects. For instance, *HDA702*/*OsHDAC1* regulates plant growth rate and alters plant architecture (Jang et al., 2003; Chung et al., 2009). Down-regulation of *HDA703/OsHDAC3* reduces rice peduncle elongation and fertility (Hu et al., 2009). *HDA710/OsHDAC2* belongs to the class I-type HDACs and is located in the opposite orientation on chromosome 2 of *japonica* rice cultivar Nipponbare (Jang et al., 2003). An RNAi mutant of *HDA710* showed severe phenotypes, including reduced vegetative growth, semi-dwarf and reduced elongation of peduncle (Hu et al., 2009). In the meanwhile, Rice HDACs play essential roles in response to stress and are related to cell death. For example, the down-regulation of HDAC *OsSRT1* affects the level of H3K9ac, activating genes associated with apoptotic cell death (Huang L. et al., 2007). Expression of both *HDA710* and *HDA703* is induced by abiotic stresses, including drought, salt, and cold stresses (Jain et al., 2007), and is also up-regulated under methylviologen (MV) treatment (Liu et al., 2010). In addition, *HDA710* and *HDA703* are preferentially expressed in Nipponbare compared to the *indica* accession 9311 (Liu et al., 2010; Jung et al., 2013). There are differences in leaf senescence between *indica* and *japonica* rice, with senescence of *indica* varieties occurring earlier than that of *japonica* varieties (Abdelkhalik et al., 2005). Leaf senescence is an important stage of plant development. In agricultural production, early leaf senescence limits the cycle of crop photosynthesis and thus affects crop yields. However, little is known about the molecular mechanism underlying the differences in leaf senescence between the rice subspecies *japonica* and *indica*.

Associations between genotype and phenotype are beginning to elucidate how genetic differences contribute to phenotypic traits. The completion of the 3,000 Rice Genomes Project (3K RGP) has provided abundant genetic resources for facilitating research establishing gene-trait associations (Li et al., 2014; Alexandrov et al., 2015). Utilization of natural genetic variation contributes greatly to improvement of important agronomic traits in crops and provides valuable resources for crop genetics and breeding improvement (Duan et al., 2017). A number of quantitative trait loci (QTLs) and genes have been identified and characterized by QTL analysis employing natural variation in rice, including traits related to flowering time (Ogiso-Tanaka et al., 2013), grain yield (Huang et al., 2009; Li Y. B. et al., 2011), and low-temperature germination (Wang X. et al., 2018).

In this study, we investigated the gene presence frequency of all 18 previously identified rice *HDAC* genes in 3,010 rice varieties using the Rice Pan-genome Browser (RPAN) and analyzed associated agricultural traits. Delay of leaf senescence by *HDA710* was further validated using transgenic approaches. To elucidate the possible regulatory mechanism of *HDA710* during leaf senescence, we used RNA sequencing (RNA-Seq), co-expression network analyses, and functional analysis of downstream genes. Our work provides a novel direction for promoting molecular crop breeding.

## Materials and Methods

### Plant Materials

Rice (Nipponbare, Zhonghua17, 9311, Teqing, and transgenic lines of *HDA710* and *OsGSTU12*) seeds were surface-sterilized in 5% (w/v) sodium hypochlorite for 20 min, washed in distilled water three or four times, then transferred to water at room temperature for 2 days followed by 37°C for 1 day to germinate. Seedlings were grown in a greenhouse (28/26°C and 12/12 h day/night) for about 2 weeks, then transplanted to natural conditions in a paddy field for phenotypic observation and experimental verification.

### Construction of Transgenic Rice Lines

Over-expression and knock-down lines of *HDA710* were generated in a Nipponbare background following a previously described method (Zhao et al., 2009), with the antisense and sense full-length CDS of *HDA710* controlled by the CaMV 35S promoter. A construct with the CaMV 35S promoter driving the CDS of *OsGSTU12* was developed for over-expression of *OsGSTU12* in Zhonghua17 rice plants. Recombinant plasmids were introduced into *Agrobacterium tumefaciens* strain EHA105 using the freeze-thaw method (Jyothishwaran et al., 2007). Transgenic rice lines were regenerated through seed-induced callus of Nipponbare or Zhonghua17, respectively (Toki et al., 2006). Transgenic plants were identified by selection with 50 mg/L hygromycin B.

### Characterization of Leaf and Whole-Plant Phenotypes

Chlorophyll content was measured in the flag leaf at the leaf tip, leaf center, and leaf base. Relative chlorophyll content was measured at multiple time points using a SPAD-502 Chlorophyll Meter, which is from Beijing Channel Science Equipment Company Limited. Phenotypic observation of fresh leaves and whole plants of each line was performed under natural conditions in the paddy fields.

### Genotype and Phenotype Association Analysis

InDels in *HDA710* and other HDACs among different rice accessions were identified from the RPAN database (http://cgm.sjtu.edu.cn/3kricedb/), which provides gene PAVs (presences and variances) for different rice accessions. The phylogenetic tree of five subspecies was also from RPAN database. The phenotypes identified for 2,266 rice accessions were downloaded from the Rice SNP-Seek Database (https://snp-seek.irri.org/_download.zul). Significance analysis of genotype and agronomic traits was performed using hypergeometric distribution, using the statistical formula displayed as follow.

$$\begin{array}{l} P=\frac{\left(\begin{array}{c} n\\ k \end{array}\right)\left(\begin{array}{c} N-n\\ K-k \end{array}\right)}{\left(\begin{array}{c} N\\ K \end{array}\right)} \end{array}$$

In the formula, *N* is the total number of all accessions, *n* is the number of accessions with deletions, *K* is the total number of accessions shown one specific phenotype and *k* is the number of accessions with deletions shown the same phenotype.

### Measurement of Chlorophyll Content

#### By Chlorophyll Meter

Chlorophyll content was measured using a SPAD-502 chlorophyll meter, which determines the relative content of the current chlorophyll of the leaf by measuring the difference in optical density at two wavelengths (650 and 940 nm) and automatically calculates the value. Each leaf blade was measured at three different locations: leaf base, leaf center, and leaf tip.

#### With Acetone Method

We took three leaves from three individual plants for each replicate to measure the chlorophyll content. Leaves were incubated in 80% acetone (v/v) for 3-6 h in the dark at 4°C then centrifuged for 10 min, 10,000 g at 4°C. Chlorophyll absorbance was measured at 646 and 663 nm, and chlorophyll contents were calculated as follows:

$$\begin{array}{l} \text{ Chlorophyll a }\left(\text{mg}/\text{mL}\right)=12.21{\text{A}}_{663}-2.81{\text{A}}_{646}\\ \text{ Chlorophyll b }\left(\text{mg}/\text{mL}\right)=20.13{\text{A}}_{646}-5.03{\text{A}}_{663}\\ \text{Chlorophyll content }\left(\text{mg}/\text{mL}\right)=\text{Chlorophyll a}+\text{ Chlorophyll b}. \end{array}$$

### Measurement of Relative Electrical Conductivity

Relative electrical conductivity was determined by measuring electrolytes leaked from leaves. Sections flag leaf of the same size were immersed in 5 mL 100 mM mannitol for 3 h with gentle shaking, after which the initial conductivity was recorded as S1. Total conductivity was determined after boiling leaves in 100°C for 10 min, and was recorded as S2. Relative electric conductivity (REC) was calculated using the following formula:

$$\begin{array}{l} \text{REC}\left(\text{\%}\right)=\left(\text{S}1/\text{S}2\right)\times 100 \end{array}$$

### RNA Isolation and qRT-PCR

All leaves were from natural conditions in paddy fields. Leaves were homogenized in liquid nitrogen and stored for a short time. Total RNA was isolated using TRIZOL® reagent (Invitrogen, CA, USA) and purified using Qiagen RNeasy columns (Qiagen, Hilden, Germany).

Reverse transcription was performed using Moloney murine leukemia virus (M-MLV; Invitrogen). A total volume of 10 μL containing 2 μg total purified RNA and 20 pmol random hexamers (Invitrogen) was heated at 70°C for 2 min and then chilled on ice for 2 min. M-MLV and the reaction buffer were added to a total volume of 20 μL containing 200 units of M-MLV, 20 pmol random hexamers, 500 μM dNTPs, 50 mM Tris-HCl (pH 8.3), 3 mM MgCl_2_, 75 mM KCl, and 5 mM dithiothreitol, and samples were then heated at 37°C for 1.5 h.

cDNA samples were diluted to 2 ng/μL for qRT-PCR assays performed on 1 μL of each cDNA dilution using SYBR Green Master Mix (Applied Biosystems, PN 4309155). The relative quantification method (ΔΔCT) was used to evaluate quantitative variation between replicates examined (Livak and Schmittgen, 2001). Primers for specific genes are listed in Table S7.

### RNA-Seq Analysis

Sequencing libraries were constructed by Annoroad (China) and sequenced using NovaSeq following standard protocols. Paired-end mRNA-Seq data consisted of read lengths of 150 bp for each sample, with three replicates for each line. Sequencing data were aligned to rice genome MSU 6.1 using Tophat v2.0.10 (Kim et al., 2013) software with default parameters. Gene expression levels represented by FPKM (fragments per kilobase of transcript per million mapped reads) were calculated by Cufflinks v2.2.1 software using default parameters (Trapnell et al., 2010). Differentially expressed genes were defined based on fold change in expression levels and q-value. If the absolute fold change was more than 1.7 and q-value <0.05, genes were considered to be differentially expressed. Gene ontology enrichment analysis was performed using agriGO (Tian et al., 2017). The gene set enrichment analysis (GSEA) was performed through the PlantGSEA website (http://structuralbiology.cau.edu.cn/PlantGSEA/).

### Immunoblots

Harvested leaves were ground in liquid nitrogen and the powder was extracted with Lysis buffer (50 mM HEPES pH 7.5, 150 mM NaCl, 1 mM EDTA, 1% TritonX-100 (v/v), 10% glycerol, 100 mM PMSF, 0.04% β-mercaptoethanol). The solution was placed on ice for 30 min and centrifuged for 20 min at 4,000 rpm at 4°C. Extracted proteins were separated on 12% SDS-PAGE gels and transferred to polyvinylidene difluoride (PVDF) membrane (Millipore, 0.22 μm). Membranes were blocked in blocking buffer [5% milk dissolved in 1 × TBST (Tris Buffered Saline with Tween 20)] at room temperature (24°C) for 1 h. Membranes were incubated overnight at room temperature with antibodies against H3K9ac (ab10812; Abcam), H3K27ac (ab4729; Abcam), and H3 (07-690; Millipore) in blocking buffer. After washing three times in TBST for 10 min each, membranes were incubated for 2 h at room temperature with horseradish peroxidase-labeled goat anti-rabbit diluted 1/5,000. Membranes were washed three times in TBST and incubated for 1 min in electrochemiluminescence (ECL) buffer (34095; ThermoFisher Scientific, http://www.thermofisher.com).

### Co-expression Network Construction

Co-expressed genes were identified for two key genes, *HDA710* and *OsGSTU12*, in the database RiceFREND (http://ricefrend.dna.affrc.go.jp). The top 300 co-expressed genes of *HDA710* sorted by Mutual rank (MR) values were downloaded. These co-expressed genes were then used to construct a co-expression network. The network was displayed by Cytoscape software.

Differentially expressed genes between *35S::HDA710-sense* and *35S::HDA710-antisense* were determined using RNA-Seq analysis. Differentially expressed genes in representatives of the two subspecies of rice, *japonica* Nipponbare and *indica* 9311 were identified from microarray data (Liu et al., 2010). All genes in the network were annotated as up-regulated, down-regulated, or showing no significant change in expression between the two genotypes.

## Results

### Investigation of InDel Polymorphisms in HDACs of Five *Oryza sativa* Subgroups

**Figure 1:**
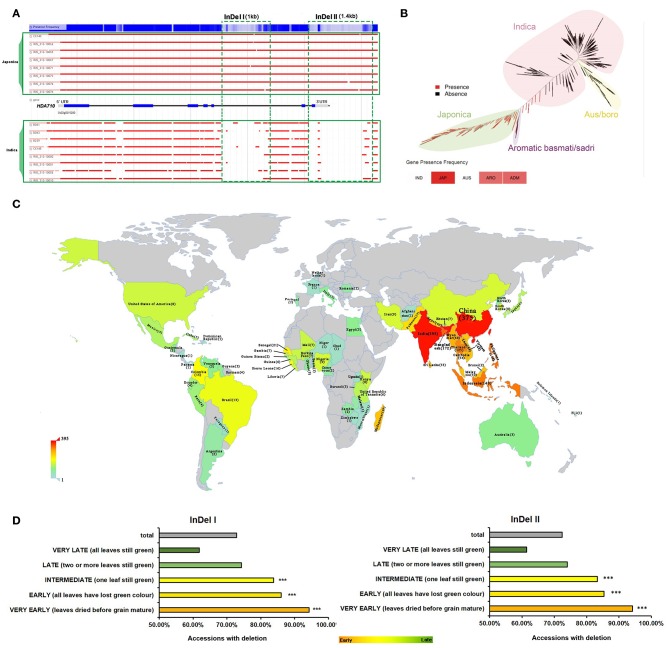
Natural variation in *HDA710* between *japonica* and *indica* accessions and association with leaf senescence. **(A)** Sequence differences in *HDA710* from *indica* and *japonica* accessions as presented by genome browser. **(B)** *HDA710* distribution among different subspecies and subgroups in the rice phylogenetic tree. **(C)** Distribution of 2,112 accessions with deletion regions (“InDel I” or “InDel II”) in *HDA710* from the 3,010 varieties at the International Rice Research Institute (IRRI). Color scale represents the number of accessions with InDels. **(D)** Leaf senescence phenotype analysis of accessions with deletions (“InDel I” and “InDel II”) by hypergeometric distribution (***P < 0.001). Different colors of bars represent degree of leaf senescence: green, accessions with late leaf senescence; yellow, accessions with early leaf senescence; gray, all accessions with deletion.

Eighteen *HDAC* genes have previously been identified in the rice genome (Hu et al., 2009). We searched for these *HDAC* genes in RPAN (http://cgm.sjtu.edu.cn/3kricedb/index.php) (Sun et al., 2017), for visual display of specific gene sequences in 3,010 rice accessions. We found significant insertion/deletion (InDel) polymorphisms in *HDA710/OsHDAC2* and *HDA703/OsHDAC3* between *japonica*-related (JAP, ARO) and *indica*-related (IND, AUS) accessions, especially for *HDA710* (Table 1). RPAN results showed that *HDA710* was present in almost 98.8% JAP accessions, and 37.6% *japonica*-like ARO accessions, while it was only present in 1% of IND accessions and 0.9% of *indica*-like AUS accessions (Table 1). The pan-genome browser based visualization of the presence/absence variation (PAVs) showed two InDels in the *HDA710* gene region, one in the gene body named “InDel I” and the other in the downstream region named “InDel II” (Figure 1A), between *indica* and *japonica* accessions. As the Table S1 shown, there were detailed information of 3010 accessions. Among of them, 2106 accessions were listed with InDel I in *HDA710*, and 2104 accessions were listed with InDel II in *HDA710*. These two InDels co-existed in 2098 (99.6%) rice accessions. We further carried out the linkage disequilibrium analysis, and the D' and r2 values were 0.9905 and 0.9780, respectively.

**Table 1:** Survey of RPAN gene presence frequency of rice histone deacetylases.

**Locus ID (MSU)**	**Locus ID (RAP)**	**Gene name**	**Gene presence frequency**				
			**JAP%**	**IND%**	**AUS%**	**ARO%**	**ADM%**
LOC_Os01g40400	Os01g0586400	HDA701	–	–	–	–	–
LOC_Os06g38470	Os06g0583400	HDA702/OsHDAC1	99.8	99.7	99.5	100	100
**LOC_Os02g12350**	**Os02g0214900**	**HDA703/OsHDAC3**	**95.8**	**28.1**	**69.7**	**82.2**	**52.8**
LOC_Os07g06980	Os07g0164100	HDA704	100	100	100	99.0	100
LOC_Os08g25570	Os08g0344100	HDA705	100	99.9	100	100	100
LOC_Os06g37420	Os06g0571100	HDA706/OsHDAC6	98.9	99.3	99.5	99.0	98.4
LOC_Os01g12310	–	HDA707	–	–	–	–	–
LOC_Os11g09370	Os11g0200000	HDA709	99.1	97.8	98.6	99.0	98.4
**LOC_Os02g12380**	**Os02g0215200**	**HDA710/OsHDAC2**	**98.8**	**1.0**	**0.9**	**37.6**	**49.6**
LOC_Os04g33480	Os04g0409600	HDA711	98.1	98.2	95.5	97.0	98.4
LOC_Os05g36920	Os05g0440250	HDA712	99.9	99.9	100.0	99.0	100.0
LOC_Os07g41090	Os07g0602200	HDA713	99.9	99.9	100.0	99.0	99.2
LOC_Os12g08220	Os12g0182700	HDA714/OsHDAC10	100	99.9	99.5	100	100
LOC_Os05g36930	Os05g0440300	HDA716	100	99.8	100	100	100
LOC_Os05g51830	Os05g0597100	HDT701	100	99.9	100	100	100
LOC_Os01g68104	Os01g0909100	HDT702	100	100	100	99.0	100
LOC_Os04g20270	Os04g0271000	SRT701/OsSRT1	100	99.9	99.1	99.0	100
LOC_Os12g07950	Os12g0179800	SRT702/OsSir2b	100	99.8	100	100	100

*Gene Presence Frequency taken from RPAN (http://cgm.sjtu.edu.cn/3kricedb/index.php). JAP, japonica; IND, indica; AUS, aus/boro group, which is more closely related to indica; ARO, aromatic basmatic/sadri group, which is more closely related to japonica; ADM, admixed, intermediate types. The bold values highlighted genes which have significant differential polymorphisms between japonica-related and indica-related accessions*.

We searched for the *HDA710* gene in RPAN to determine the distribution of this gene among 453 high-quality accessions, finding that *HDA710* was dominant in *japonica* accessions and also present in *japonica*-like ARO and intermediate type ADM accessions (Figure 1B) (Sun et al., 2017). We also found that *HDA703* differed among the five rice subgroups, being present in 95.8% *japonica* accessions and 82.2% ARO accessions, but only 28.1% *indica* accessions (Table 1).

To verify whether there are also differences in the expression of *HDA710* and *HDA703* between *indica* and *japonica*, we investigated microarray data for *japonica* rice cultivar Nipponbare and *indica* rice accession 9311 (Liu et al., 2010). The microarray data showed higher expression levels of *HDA710* in Nipponbare than in 9311 (Table S2). Data from 983 Affymetrix microarrays from *japonica* and *indica* confirmed that *HDA710* is highly expressed in *japonica* (Jung et al., 2013). The microarray data also indicated that *HDA703* is highly expressed in Nipponare compared to 9311 (Table S2).

There were no significant differences in gene presence frequency for the other HDACs (Table 1). Based on the differences in genotype and expression profile of *HDA710* compared with the other deacetylases, we proposed that *HDA710* might be a key gene for the differentiation of *indica* and *japonica* rice in plant growth and development and stress resistance.

### *HDA710* Is Associated With Leaf Senescence by Gene-Trait Association Study

Through association analysis between genotypic diversity and corresponding phenotypic diversity, we can predict how the genotype contributes to the phenotype (Mansueto et al., 2017). The 3K RGP accessions were classified into different subpopulations, most of which could be connected to geographic origins (Wang W. et al., 2018). To explore whether the accessions with deletions were connected with the global climate, we examined the geographical distribution of 2,112 accessions harboring deletions in *HDA710* (Figure 1A and Table S1) from the 3,010 accessions at the International Rice Research Institute (IRRI). We found that these accessions were mainly distributed in tropical and subtropical regions (Figure 1C). In addition to sequence information, more detailed phenotypic data (http://snp-seek.irri.org/download.zul) were available for 2,266 of these 3,010 accessions, allowing us to conduct a gene-trait association study. We examined the relationship between “InDel I”/“InDel II” (Figure 1A and Table S1) and multiple agronomic traits of the accessions, including panicle exsertion, awn presence, culm length, panicle threshability, seedling height, culm number, flag leaf angle, and leaf senescence. Leaf senescence was the most significant trait associated with genotype diversity. Some other agronomic traits were also associated. As shown in Figure S1, accessions with deletions exhibited partial panicle exsertion, awn absence, long culms, prolific culm number, easy panicle threshability, and erect flag leaves. Association analysis between the agronomic trait “leaf senescence” and the accessions with deletions showed that the 1,647 accessions with absence of the “InDel I” fragment tended to exhibit very early leaf senescence (Figure 1D and Table S1), as did the 1,639 varieties with absence of the “InDel II” fragment. Thus, our results indicated that *HDA710* possesses natural genotypic variation between *indica*-related and *japonica*-related accessions, which was significantly associated with leaf senescence.

### Generation of Transgenic Rice Lines Reveals *HDA710* as a Negative Regulator of Leaf Senescence

**Figure 2:**
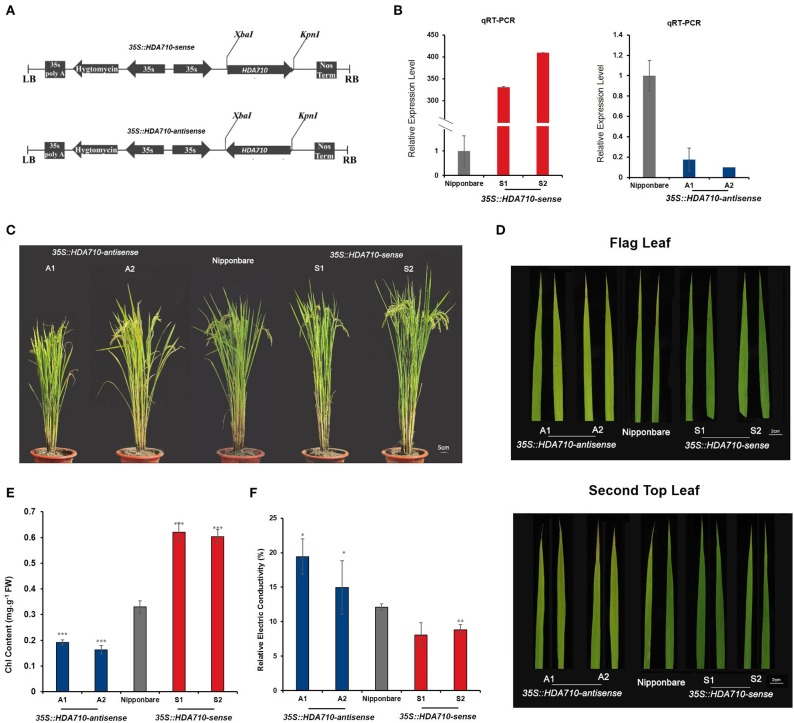
Phenotypic observation of leaf senescence at the late developmental growth stage in *HDA710* transgenic plants and controls. **(A)** Vectors *35S:HDA710-sense* and *35S:HDA710-antisense*. **(B)** qRT-PCR validation of relative *HDA710* expression levels in *35S:HDA710-antisense*, Nipponbare, and *35S:HDA710-sense* plants. Data are means ± SD (*n* = 3). **(C)** Whole plants of *35S:HDA710-antisense*, Nipponbare, and *35S:HDA710-sense* taken from the field at about 104 days. Bar = 5 cm. **(D)** Mature leaf before harvest in *35S:HDA710-antisense*, Nipponbare, and *35S:HDA710-sense*. Bar = 2 cm. **(E)** Chlorophyll content of second top leaves of *35S::HDA710-antisense*, Nipponbare, and *35S::HDA710-sense* plants. We performed six biological replicates, each of which was derived from three leaves in three individual plants. **(F)** Relative electrical conductivity in flag leaves at about 114 days. We performed four biological replicates, each of which was derived from four leaves in four individual plants. One-way ANOVA results for genotypic variation comparisons in **(E,F)** are given in Table S3. The error bars in **(B,E,F)** represent the standard error of replicates. The asterisks represent the significant difference between Nipponbare, *35S::HDA710-antisense*, and *35S::HDA710-sense* (*P < 0.05; **P < 0.01; ***P < 0.001).

Correlation analysis between genotypes and phenotypes of various accessions revealed that the deletion in *HDA710* might activate the early leaf senescence process. We used an antisense and sense transgenic approach to characterize the function of the *HDA710* gene in rice plants. The cloned full-length coding sequence (CDS) of *HDA710* was used to generate transgenic rice lines with *Oryza sativa* ssp. *japonica* cv. Nipponbare as the wild-type (WT) background. Down-regulation of *HDA710* was achieved by an antisense approach (Figure 2A) and over-expression by a sense approach (Figure 2A) under control of the 35S promoter.

We determined expression levels of *HDA710* in Nipponbare (WT) and transgenic rice lines by quantitative reverse-transcription PCR (qRT-PCR) using specific primers for *35S::HDA710-antisense* and *-sense* transgenic lines. Relative expression levels of *HDA710* were significantly down-regulated in the *35S::HDA710*-*antisense* lines and significantly higher than those in Nipponbare in the *35S::HDA710-sense* lines (Figure 2B).

To further explore the relationship between *HDA710* expression levels and histone deacetylation activity, we performed immunoblotting with specific antibodies. We detected acetylation of histone H3 at lysine 9 and 27 (H3K9ac and H3K27ac) in *35S::HDA710-antisense* and -*sense* transgenic lines, *japonica* rice Nipponbare. As shown in Figure S2, the two antisense lines had higher levels of H3K9ac and H3K27ac than Nipponbare and the *35S::HDA710-sense* lines.

To investigate leaf senescence during plant growth stages, *35S::HDA710*-*sense, 35S::HDA710*-*antisense*, and Nipponbare (WT) plants were planted in paddy fields. Leaf yellowing is a convenient visible characteristic of leaf senescence, mainly reflecting the chloroplast senescence of mesophyll cells and chlorophyll loss (Oh et al., 1997). We investigated leaf senescence in whole plants, observing that the over-expression lines stayed green compared with the knock-down lines and Nipponbare (Figure 2C). *35S::HDA710-antisense* lines showed earlier senescence than Nipponbare in the flag leaves and second top leaves; in contrast, leaves of *35S::HDA710-sense* lines stayed green (Figure 2D). The second top leaves of over-expression lines had a higher chlorophyll content than those of Nipponbare, while leaves of the knock-down lines showed a slightly lower chlorophyll content (Figure 2E and Table S3). We also measured relative electrical conductivity, another senescence-related indicator. When leaves grow older, membranes become fragile, and electrolytes leak out of cells (Chen et al., 2015). Ion leakage was higher in *35S::HDA710-antisense* lines than in *35S::HDA710-sense* lines or Nipponbare (Figure 2F and Table S3).

To investigate the specific time of leaf senescence during plant growth, we tested *35S::HDA710*-*sense* lines, *35S::HDA710*-*antisense* lines, and Nipponbare (WT) plants at different stages (Figure S3). In the field, we used the SPAD-502 chlorophyll meter to measure the chlorophyll content in six time points (from 75d to 118d after planting). Each time point we took more than 10 leaves from individual plants. We started to examine chlorophyll content at 75 days after planting, and all results showed decreasing chlorophyll content over time (Figure S3). The knock-down *35S::HDA710*-*antisense* lines had a relatively lower chlorophyll content than Nipponbare; while, the over-expression *35S::HDA710*-*sense* lines showed a relatively higher chlorophyll content than the WT, especially after 100 days (Figure S3).

We also measured chlorophyll content in the leaf tip, leaf center and leaf base of *japonica* rice Nipponbare and *indica* accessions 9311 and Teqing (TQ). Nipponbare had a higher chlorophyll content than 9311 or TQ, suggesting that *japonica* indeed shows a late leaf senescence phenotype (Figures S4A,B). The *indica* 9311 and TQ accessions also tended to have higher relative electrical conductivity (Figure S4C).

### RNA-Seq Based Transcriptome Analysis of *HDA710* Transgenic Lines and Nipponbare

To explore the mechanism of *HDA710* regulation of leaf senescence, we applied a transcriptomic strategy using RNA-Seq to investigate differences in whole-genome gene expression among *35S::HDA710-sense*, Nipponbare, and *35S::HDA710-antisense*. We used flag leaves of these lines with three independent biological replicates for sequencing. All RNA-Seq reads were aligned to the rice genome version MSU6.1 using Tophat (Kim et al., 2013), with alignment rates for all the samples at around 90% (Table S4). Using Cufflinks, we identified differentially expressed genes between *35S::HDA710-sense* and *35S::HDA710-antisense* with a cut-off value fold change ≥1.7 and q-value ≤0.05. We identified 2,809 differentially expressed genes, including 1,664 significantly up-regulated genes in *35S::HDA710-sense* and 1,145 significantly down-regulated genes in *35S::HDA710-sense* compared with *35S::HDA710-antisense*, respectively (Figure S5A).

In addition to the differentially expressed genes between antisense and sense lines, we also calculated the differentially expressed genes (DEGs) of *35S::HDA710-sense* vs. Nipponbare and *35S::HDA710-antisense* vs. Nipponbare. The detail numbers about up- and down- regulated genes were shown in Figure S5A. In the meanwhile, we performed SEACOMPARE analysis using agriGO webserver and the results showed that, the GO terms such as “photosynthesis” and “chlorophyll biosynthetic” were significantly enriched in the genes up-regulated in both Nipponbare and *35S::HDA710-sense* line compared to *35S::HDA710-antisense*; while the GO terms such as “programmed cell death,” “apoptosis” and “response to stress” were enriched in the genes down-regulated in the *35S::HDA710-sense* line compared to Nipponbare and *35S::HDA710-antisense* (Figure S5B).

**Figure 3:**
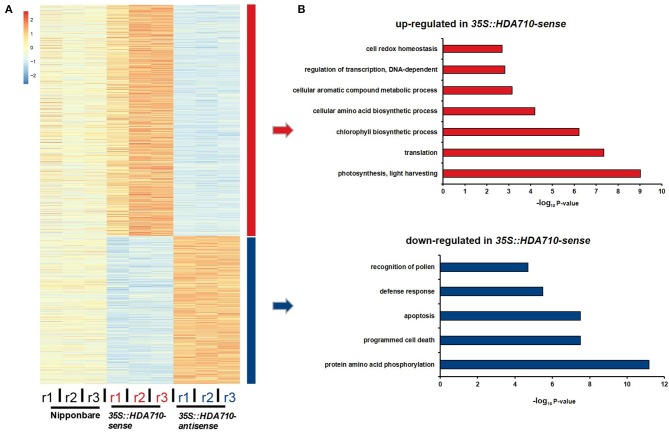
RNA-Seq analysis of *35S::HDA710-antisense* and *35S::HDA710-sense*. **(A)** Heatmap showing relative expression levels of differentially expressed genes in different replicates of each sample. **(B)** GO term enrichment analysis corresponding to the up-regulated and down-regulated genes in **(A)**.

To identify co-expressed genes with similar expression patterns in transgenic and Nipponbare plants, we employed the hierarchical method to cluster the 2,809 differentially expressed genes between *35S::HDA710-antisense* and *35S::HDA710-sense*. From the heat map representing relative expression level of each gene across the nine samples, these genes were obviously clustered into two groups, one representing genes with higher expression in *35S::HDA710-sense* plants and another representing genes with higher expression in *35S::HDA710-antisense* plants (Figure 3A).

**Figure 4:**
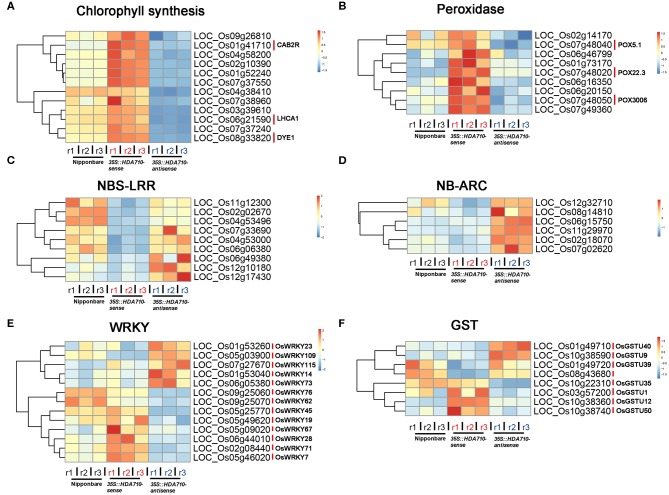
Expression patterns of gene superfamilies in Nipponbare, 3*5S::HDA710-sense*, and *35S::HDA710-antisense*. Genes derived from Chlorophyll biosynthesis **(A)**, Peroxidase **(B)**, NBS-LRR **(C)**, NB-ARC **(D)**, WRKY **(E)**, and GST **(F)** families. Red indicates a higher expression level, whereas blue indicates a lower expression level.

We conducted gene GO enrichment analysis for these differentially expressed genes in *35S::HDA710-sense* vs. *35S::HDA710-antisense*. For genes up-regulated in *35S::HDA710-sense*, the GO terms “photosynthesis, light harvesting” and “chlorophyll biosynthetic process” were significantly enriched (Figure 3B). Twelve genes related to chlorophyll biosynthesis were highly expressed in *35S::HDA710-sense* compared to Nipponbare and *35S::HDA710-antisense* (Figure 4A and Table S5). The GO term “cell redox homeostasis” was also enriched (Figure 3B). Reactive oxygen species (ROS) are important in early leaf senescence (Allu et al., 2014); therefore, we investigated the differentially expressed peroxidase genes and found that nine of them were preferentially expressed in the *35S::HDA710-sense* line (Figure 4B and Table S5). Among down-regulated genes in *35S::HDA710-sense*, the GO terms “cell death,” “apoptosis,” and “defense response” were significantly enriched (Figure 3B). This indicates that leaf senescence is accompanied by cell death and triggers the expression of some defense response genes. Nucleotide-binding site leucine-rich repeat (NBS-LRR) proteins are involved in the detection of diverse pathogens, including bacteria, viruses, fungi, nematodes, insects, and oomycetes (McHale et al., 2006). The NB-ARC domain is a functional ATPase domain, and its nucleotide-binding state is proposed to regulate activity of the R protein (van Ooijen et al., 2008). Interestingly, these two kinds of R genes were both up-regulated in *35S::HDA710-antisense* (Figures 4C,D and Table S5).

We also conducted gene set enrichment analysis (GSEA) through literature mining, which showed that the 2,809 differentially expressed genes were significantly enriched in the “BTH (benzothiadiazole)-inducible and WRKY45-dependent” gene set (Nakayama et al., 2013), with *p*-value = 5.8e−16 (Figure S6). Among the reported 277 BTH-inducible and WRKY45-dependent rice genes, there were 51 differentially expressed genes (28 up-regulated and 23 down-regulated genes in *35S::HDA710-sense* vs. *35S::HDA710-antisense*), including *WRKYs, HDA710*, and *glutathione S-transferase* (*GST*) genes (Figure S6). The *GST* gene family encodes genes critical for certain life processes and mechanisms of detoxification and toxicity (Nebert and Vasiliou, 2004). We also mined data from the expression profiles of the differentially expressed *WRKY* and *GST* family genes (Figures 4E,F and Table S5). *WRKY* and *GST* family genes showed some similar expression patterns, and both of them were regulated by *HDA710*. For example, Os*WRKY45* and Os*WRKY62* as well as Os*GSTU1*, Os*GSTU12*, Os*GSTU35*, and Os*GSTU50* were up-regulated in the *35S::HDA710-sense* lines (Figures 4E,F and Table S5). Interestingly, these *WRKY* and *GST* genes were also included in the list of 277 BTH-inducible and WRKY45-dependent rice genes (Figure S6).

### Co-expression Network of *HDA710* Reveals Synergistic Roles of *HDA710* and Os*GSTU12* in Regulating Leaf Senescence

Construction of a co-expression gene network is an efficient method for determining linkages between genes in a biological process. We acquired the top 300 genes positively co-expressed with *HDA710* from RiceFREND (Table S6), a gene co-expression database in rice (Sato et al., 2013), and further found that *GST* family genes were enriched in GSEA analysis of these genes using the PlantGSEA platform (Yi et al., 2013). This suggested that *GST* genes might be induced in the same pathways or by similar factors as *HDA710*. We focused on the differentially expressed genes in *35S::HDA710-sense* vs. *35S::HDA710-antisense* RNA-Seq profiles as candidates to test whether *GST* genes share a common function with *HDA710*. Eleven genes overlapped with the top 300 co-expressed genes of *HDA710* up-regulated in *35S::HDA710-sense* RNA-Seq profiles; four of these were also up-regulated in *japonica* Nipponbare compared with *indica* 9311 from microarray data (Liu et al., 2010), including *OsGSTU12* (*LOC_Os10g38360*), *LOC_Os01g67860, LOC_Os01g15270*, and *LOC_Os10g02380* (Table S6). Thus, these four key candidates potentially play a similar role in various biological pathways and/or metabolic processes, especially leaf senescence.

**Figure 5:**
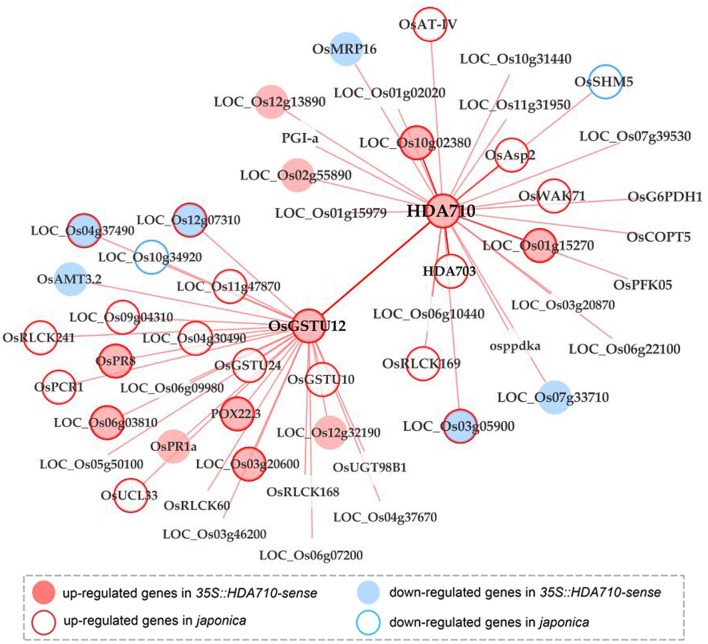
Co-expression network of *HDA710* combined with transcriptome data reveals a close link with *OsGSTU12*. The network is constructed from RiceFREND using Cytoscape software. Nodes in light pink represent genes up-regulated in *35S::HDA710-sense* vs. *35S::HDA710-antisense*, while nodes in light blue represent genes down-regulated in *35S::HDA710-sense* vs. *35S::HDA710-antisense*. Nodes with light pink borders represent genes up-regulated in *japonica* Nipponbare vs. *indica* 9311, while nodes with light blue borders represent genes down-regulated in *japonica* Nipponbare vs. *indica* 9311. Lines between nodes represent positive co-expression relationships.

To further analyze the genetic diversity of the four genes, we identified InDels in the promoter region of the *OsGSTU12* gene in 3,010 accessions from different rice subspecies using the RPAN database (Figure S7). We found a deletion of around 1 kb in the promoter region of *OsGSTU12* in majority of *indica* accessions compared with *japonica* accessions. InDels in the promoter region of *OsGSTU12* and in the gene region of *HDA710* occurred coincidentally among more than 90% of the 3,010 rice accessions (Table S1). Interestingly, *OsGSTU12* and *HDA710* are both induced by *OsWRKY45* (Nakayama et al., 2013) (Figure S6), suggesting that *OsGSTU12* and *HDA710* are both regulated by *OsWRKY45*. As the *OsWRKY45* transcription factor regulates genes through W-box motifs (Nakayama et al., 2013; Fukushima et al., 2016), we investigated W-box motifs in the promoter region of *OsGSTU12*. We identified 21 W-box motifs in the promoter region of *OsGSTU12*, especially in the InDel region (Figure S7). We also searched for genes co-expressed with *HDA710* and *OsGSTU12*, and constructed a co-expression gene network with different expression views (Figure 5). Five genes co-expressed with *HDA710*, including *OsGSTU12*, were up-regulated in *35S::HDA710-sense* lines, and meanwhile six genes co-expressed with *OsGSTU12* were also up-regulated in *35S::HDA710-sense* lines compared with *35S::HDA710-antisense* lines. Furthermore, *HDA710* together with nine co-expressed genes, including *OsGSTU12*, were up-regulated in *japonica* rice Nipponbare vs. *indica* rice 9311. Additionally, *OsGSTU12* and 14 co-expressed genes showed higher expression levels in Nipponbare than in 9311. Consistency in sequence variations and co-expression networks between *HDA710* and *OsGSTU12* implied common involvement in some subspecies-specific biological pathways. Our results suggest that *HDA710* and *OsGSTU12* possibly work synergistically in the regulation of leaf senescence.

### Over-Expression of *OsGSTU12* in Rice Delays Leaf Senescence

**Figure 6:**
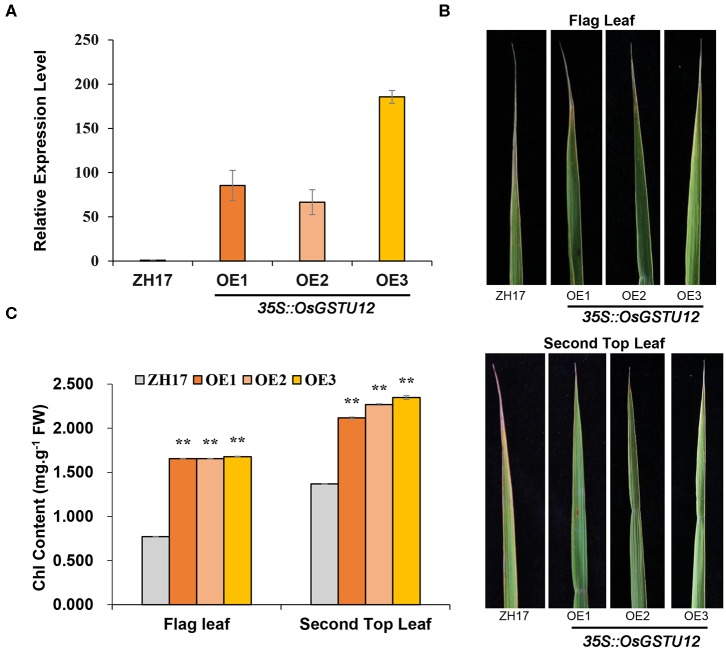
Comparative analysis of transgenic lines over-expressing *OsGSTU12* with controls at the late developmental growth stage in the field. **(A)** Relative expression levels of Os*GSTU12* in the wild type (ZH17) and *OsGSTU12* over-expression transgenic lines (OE1, OE2, OE3). Data are means ± SD (*n* = 3). **(B)** Flag leaf and second top leaf in wild-type and over-expression lines. **(C)** Chlorophyll content of flag leaf and second top leaf from top. Data are means ± SD (*n* = 4). **P < 0.01; Student's *t*-test.

Since *OsGSTU12* is highly expressed in *japonica* rice, similar to *HDA710*, and was co-expressed with *HDA710* during *HDA710*-mediated leaf senescence (Figure 5), we over-expressed *OsGSTU12* in rice cultivar Zhonghua17 to determine whether over-expression of *OsGSTU12* could prolong the natural aging of rice. Three over-expression transgenic lines (OE1, OE2, OE3) showing higher relative *OsGSTU12* expression levels than wild-type Zhonghua17 were observed in the field at the grain-filling stage (Figure 6A). As shown in Figure 6B, the top parts of flag leaves and second top leaves of Zhonghua17 had begun to yellow and whither, but those of over-expression lines retained considerably more green parts. Further chlorophyll content analysis showed that the flag leaves and second top leaves of over-expression lines had significantly higher chlorophyll content compared to that of Zhonghua17 (Figure 6C), suggesting that over-expression of *OsGSTU12* could delay the natural leaf senescence of rice at the late developmental growth stage.

## Discussion

### *HDA710* Shows Natural Variation Between *Oryza sativa* ssp. *japonica* and *indica*

Natural variation reflects sequence differences introduced during long-term evolution of species due to various environmental factors, resulting in phenotypic differences. Although the genome-wide association study (GWAS) approach is popular for mining important agronomic traits in crops, it remains a slow process. Previous studies have reported differences in the sequence and gene expression of *HDA710* in *indica* and *japonica* rice (Liu et al., 2010). Our qRT-PCR experiment also demonstrated higher expression levels of this gene in Nipponbare than in the *indica* accession 9311. Examination of gene sequence differences in representatives of different subspecies in the RPAN database revealed that *HDA710* was *japonica*-dominant, with two fragments absent mainly in *indica* rice accessions, namely “InDel I” and “InDel II” regions. Differences in genotype are often an important factor leading to phenotypic differences, so the absence of these two fragments may be responsible for differences in the traits of *japonica* and *indica* rice. By comparing genotypes and phenotypes, we found that accessions with missing fragments were more inclined to early senescence of leaves. The *indica* rice accessions showed a yellowing phenotype and lower chlorophyll content than *japonica* accessions. Since most of the accessions with missing *HDA710* fragments belonged to the *indica* subspecies, whereas the sequence of this gene was relatively complete in *japonica* rice, *HDA710* represents an important marker for *indica* and *japonica* differentiation. In summary, we suggest that the natural variation of *HDA710* may be key for leaf senescence traits. As it is present in only 1% *indica* accessions, it may have great potential for introduction into other *indica* rice accessions.

### *HDA710* May Regulate Leaf Senescence Through Programmed Cell Death and Free Radical Reaction

Leaf senescence is a complex trait involved in various biological processes, including degradation of chloroplasts, degradation of nucleic acids and proteins, and recycling of nutrients (Zeng et al., 2018). Early leaf senescence has a negative effect on rice yield and quality; therefore, it is of great importance to explore the hidden molecular mechanisms.

Transcriptome data analysis indicated that *HDA710* regulates many genes associated with leaf senescence. When *HDA710* gene expression was up-regulated, it activated genes related to chlorophyll biosynthesis, photosynthesis, and light harvesting. We analyzed some key gene families and found high expression levels of many chlorophyll biosynthesis related genes in *HDA710* over-expression lines showing late leaf senescence, which directly affected the color of leaves. Conversely, down-regulation of *HDA710* gene expression induced expression of defense responses and apoptosis-related genes, with some GO terms related to cell death significantly enriched. Genes related to these GO terms mostly belonged to NBS-LRR and NB-ARC families, reported to be mainly involved in diseases resistance. Cell death and disease resistance are closely linked in plants. In fact, cell death occurring in leaf senescence is a type of programmed cell death (PCD) controlled by many active genetic programs (Cao et al., 2003). Pathogenesis-related (PR) proteins are involved in PCD, and many PR genes are induced during leaf senescence in plants (Quirino et al., 1999, 2000). There is already ample evidence suggesting that cell death reinforces or stimulates the induction of plant defense processes (Shirasu and Schulze-Lefert, 2000). In addition, up-regulation of *HDA710* expression induces high expression levels of some peroxidases that inhibit free radical production. Scavenging of free radicals blocks the activity of reactive oxygen species, thereby protecting cells and tissues from oxidative damage, which plays an important role in delaying leaf senescence.

Studies have shown that leaf senescence may be caused by the destruction of chloroplasts, leading to a decrease in metabolic rate and thereby reducing the effects of oxidative stress (Woo et al., 2002). Production of ROS is one of the earliest reactions of plant cells under abiotic stress and senescence processes (Khanna-Chopra, 2012). In *Arabidopsis*, PCD, developmental senescence, and resistance response are connected through complicated genetic controls influenced by redox regulation (Pavet et al., 2005). We analyzed MV-treatment microarray data and found that *HDA710* and *OsGSTU12* might be involved in the response to oxidative stress (Liu et al., 2010). This suggests that *HDA710* may play a role in regulating leaf senescence by scavenging free radicals together with *OsGSTU12*. In order to verify the differential expression of *HDA710* between the two rice genotypes and whether it is capable of responding to oxidative stress, we treated Nipponbare and 9311 with MV for 6 and 24 h. qRT-PCR showed higher expression levels of *HDA710* in Nipponbare than in 9311, which were significantly induced after MV treatment (Figure S8). We demonstrated here that *HDA710* is involved in cell death, PR, and oxidative stress during leaf senescence.

### *HDA710* and *OsGSTU12* Exhibit Possible Co-regulatory Mechanisms and May Work Synergistically in Regulating Leaf Senescence in Rice

Most agronomic traits are regulated by multiple genes and various pathways (Rao et al., 2014). Co-expressed genes are those with similar trends of gene expression in the same tissue or under the same stress treatment conditions, and these genes usually participate in a defined biological process. Induction or suppression of *HDA710* resulted in differential expression of a large number of genes, some showing strong co-expression relationships, suggesting that these genes may take part in the same molecular process of regulating leaf senescence. One of the key genes, *OsGSTU12*, was up-regulated in *35S::HDA710-sense* lines vs. *35S::HDA710-antisense* lines and positively regulated by *HDA710*. Expression of *OsGSTU12* was higher in *japonica* rice Nipponbare compared to *indica* rice 9311. Most of the genes co-expressed with *OsGSTU12* were up-regulated in Nipponbare, suggesting that *OsGSTU12* and its co-expressed genes might be similarly affected by changes in the expression of *HDA710*, possibly working in coordination with *HDA710* in delaying rice leaf senescence. Consistent with the co-expression network analyses, we indeed found that over-expression of *OsGSTU12* could delay the process of leaf senescence (Figure 6).

In addition, the promoter region of *OsGSTU12* shows natural variation between the *indica* and *japonica* rice subspecies. Coincidentally, the presence/absence variation (PAVs) of *OsGSTU12* was consistent with that of *HDA710* in more than 90% of 3,010 rice accessions (Table S1), hinting that *HDA710* and Os*GSTU12* had a co-evolutionary relationship during the divergence of rice subspecies.

**Figure 7:**
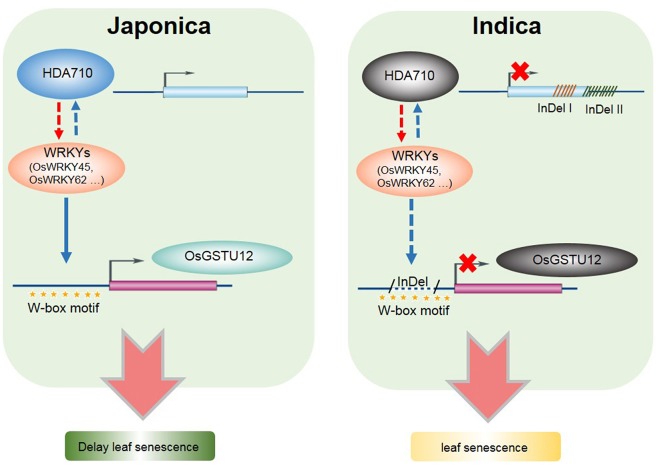
Model of *HDA710* working synergistically with *OsGSTU12* in regulating the process of leaf senescence in rice. Here presented a pathway of regulating leaf senescence in *indica* and *japonica* rice. The red arrow represents the *HDA710* gene could regulate the expression of *WRKYs* through the RNA-Seq data, while the blue arrow represents the *WRKYs* could regulate the expression of *HDA710* and *OsGSTU12* though the published data. In *indica* rice, the gray oval which represent the gene *HDA710* and *OsGSTU12* means that these two genes are down-regulated compared to *japonica* rice, and both of these two genes showed deletions. In *japonica* rice, there are W-box binding sites, while in *indica* rice, there are deletions which cover partial W-box binding sites.

We propose a model in which *HDA710* works synergistically with *OsGSTU12* in regulating the process of leaf senescence in rice (Figure 7). Both *HDA710* and *OsGSTU12* may be regulated by specific *OsWRKYs* (such as *OsWRKY45* and *OsWRKY62*), which are in turn induced by over-expression of the *HDA710* gene. These two genes exhibit InDels between *indica* and *japonica* rice accessions. In *japonica* rice, *HDA710* promotes expression of these *OsWRKYs*, which further bind to the W-box motif of *OsGSTU12*, thereby activating expression of *OsGSTU12* and delaying leaf senescence; in *indica* rice, the *HDA710* gene has fragment deletions in the gene region, and *HDA710* expression is inhibited, thereby down-regulating expression of these *OsWRKY*s. Furthermore, in *indica* rice, deletion of the promoter region of *OsGSTU12* may affect binding of OsWRKYs to the W-box motif, thereby preventing *OsGSTU12* from being activated and leading to early leaf senescence. This may partially elucidate the molecular mechanism of earlier leaf senescence in *indica* rice accessions compared to *japonica* rice accessions. The specific relationships among *HDA710, OsWRKYs*, and *OsGSTU12* need to be further explored, and proteins interacting with HDA710 as well as its downstream target genes should also be further analyzed.

## Conclusion

In summary, we systematically studied the association of *HDA710* with leaf senescence in rice. We surveyed the gene presence frequency of all rice *HDAC* genes in 3,010 rice accessions, determining that deletions of fragments of *HDA710* are significantly involved in the process of leaf senescence. Further transgenic results validated that *HDA710* represses leaf senescence in rice. We also conducted RNA-Seq analysis of the transgenic lines to elucidate the possible regulation mechanism and further constructed a co-expression network for *HDA710*, which revealed that the *GST* gene *OsGSTU12* is co-expressed and possibly co-evolved with *HDA710* for regulating leaf senescence in rice. Further transgenic analysis showed that over-expression of *OsGSTU12* also delayed leaf senescence in rice. We expect that epigenetic and genetic variation in diverse accessions will provide valuable resources for improving important agronomic traits, and widen sources of phenotypic variation in crops. The association of histone deacetylase *HDA710* with leaf senescence in rice will provide a novel direction for promoting the application of epigenetic and genetic variation together with co-expression networks in crop breeding.

## Data Availability Statement

The datasets generated for this study can be found in the http://bioinformatics.cau.edu.cn/HDA710_RNA-seq/.

## Author Contributions

WX, ZS, and FL: conceived and designed the experiments. NZ, MS, JZ, WX, QW, QS, KZ, and FL: performed the experiments. NZ, XM, WX, ZS, CS, and FL: analyzed the data. XM and ZS: contributed bioinformatics platform and analysis tools. WX, NZ, JZ, XM, FL, and ZS: wrote the paper.

## Conflict of Interest

The authors declare that the research was conducted in the absence of any commercial or financial relationships that could be construed as a potential conflict of interest.
