# Supplementary material for: Over-Expression of HDA710 Delays Leaf Senescence in Rice (Oryza sativa L.)
Source: Front Bioeng Biotechnol. 2020 May 19;8:471. doi: 10.3389/fbioe.2020.00471 (PMC7248171; doi:10.3389/fbioe.2020.00471)
Supplement: Supplementary file 5 [file Data_Sheet_1.pdf]

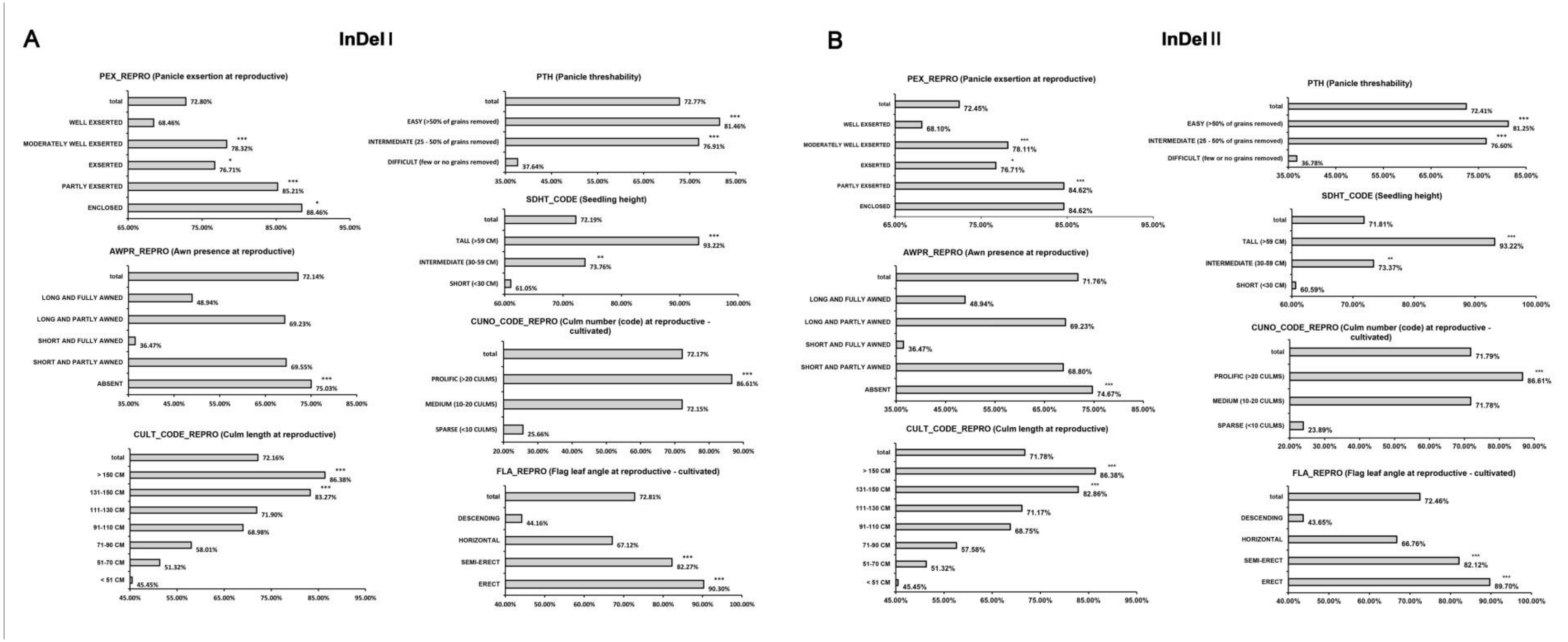

Figure S1. Phenotype analysis of varieties with InDel I (A) and InDel II (B) in *HDA710* by hypergeometric distribution (*P<0.05; **P<0.01; ***P<0.001).

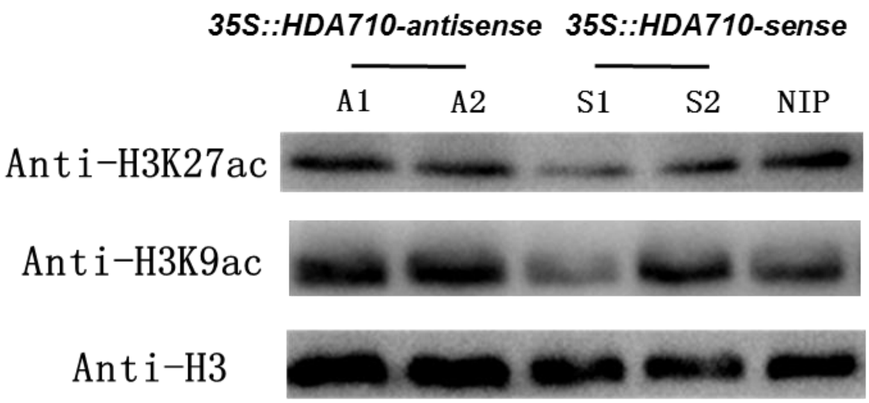

Figure S2. Immunoblot to detect H3K9ac and H3K27ac levels in *35S::HDA710-antisense*, *35S::HDA710-sense*, Nipponbare. Protein loading was assessed using an antibody that recognizes histone H3.

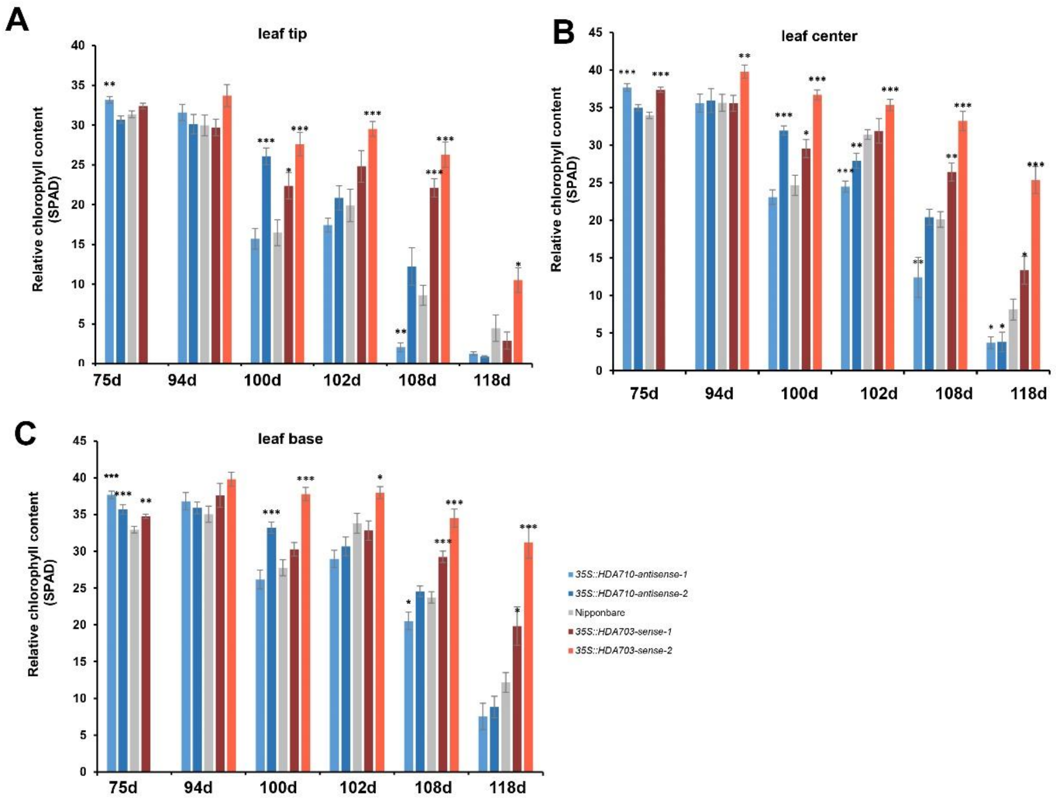

Figure S3. Chlorophyll content of leaf tip, leaf center and leaf base of flag leaves. Measurement of chlorophyll content in leaf tips (**A**), leaf center (**B**) and leaf base (**C**) of flag leaves at different growth stages. The asterisks reoresent the significant significant difference of Nipponbare, *35S::HDA710-antisense*, and *35S::HDA710-sense* (*P<0.05; **P<0.01; ***P<0.001).

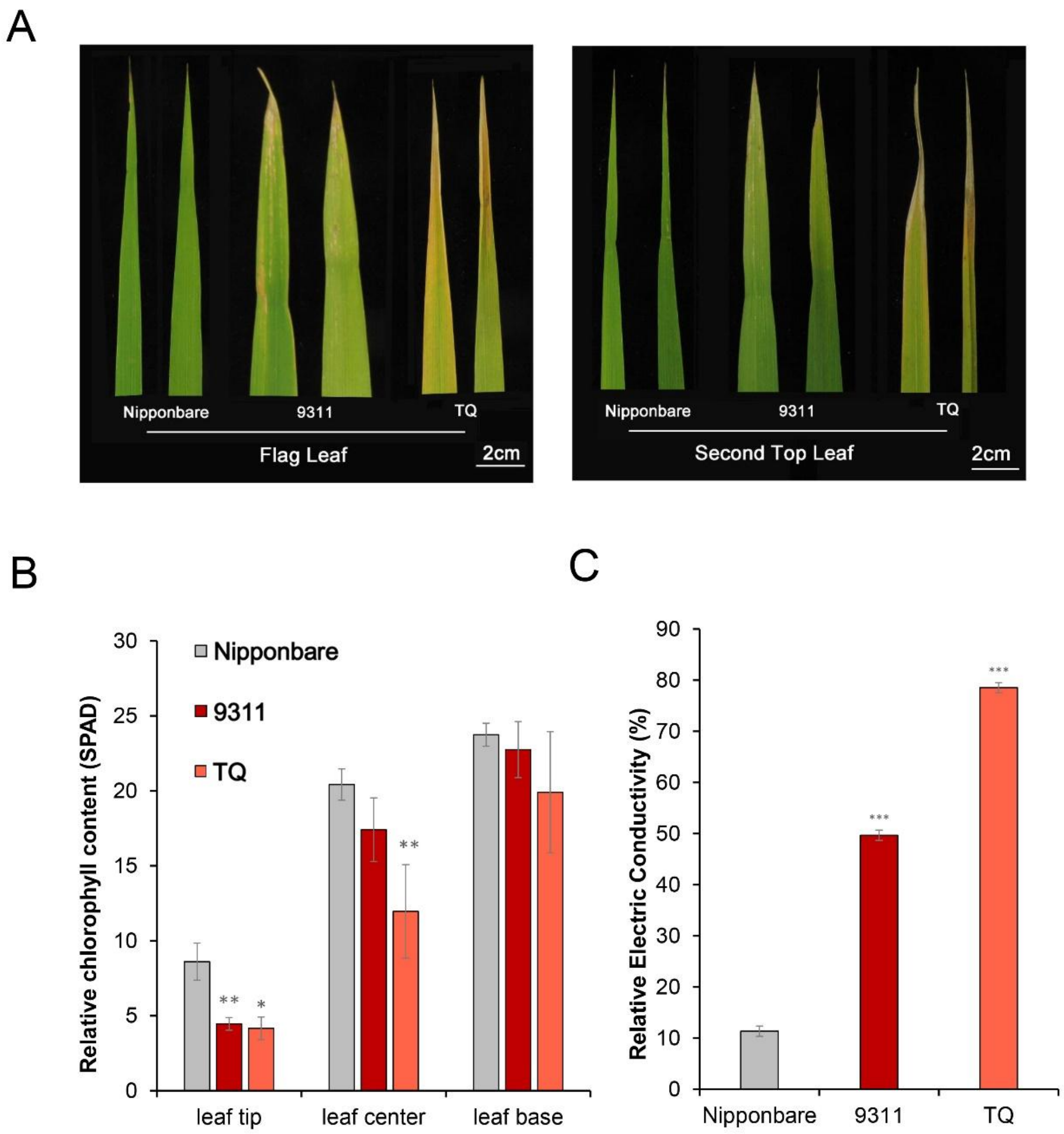

Figure S4. Phenotypic analysis of Nipponbare, 9311, and TQ.

(A) Flag leaf and the second top leaf of Nipponbare, 9311 and TQ. **(B)** Relative chlorophyll content in leaf tip, leaf center and leaf base. **(C)** Relative electric conductivity of Nipponbare, 9311, and TQ flag leaves at 114 days. The error bars in (**B**) and (**C**) represent the standard error of replicates. The asterisks represent the significant difference between Nipponbare, 9311, and TQ (*P<0.05; **P<0.01; ***P<0.001).

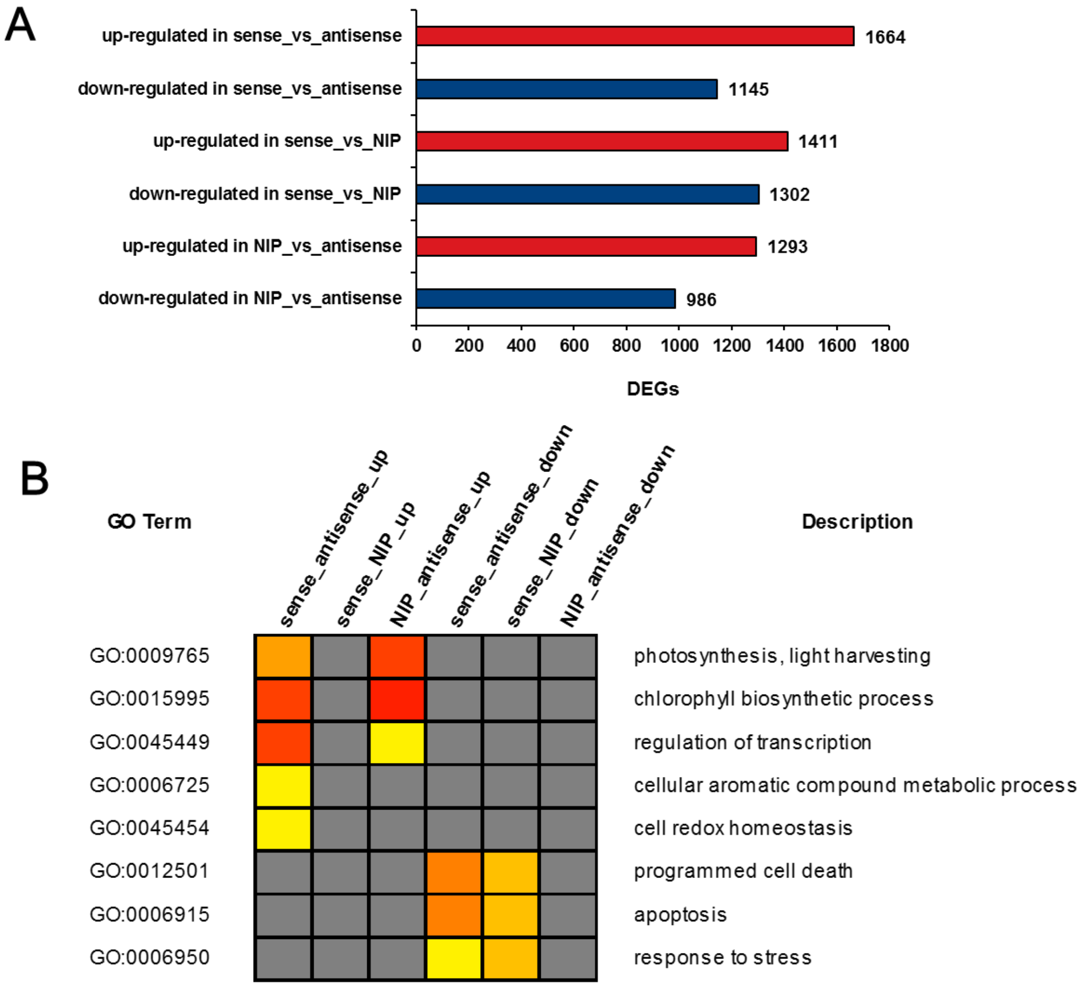

Figure S5. Comparative analysis of the differentially expressed genes among *35S::HDA710-sense*, *35S::HDA710-antisense*, and Nipponbare (WT).

(A) Up-regulated and down-regulated genes in *35S::HDA710-sense* compared with *35S:: HDA710-antisense*, *35S::HDA710-sense* compared with Nipponbare, and Nipponbare compared with *35S:: HDA710-antisense* (fold change >1.7, q-value <0.05). **(B)** SEACOMPARE analysis of the differentially expressed genes (by agriGO v2.0).

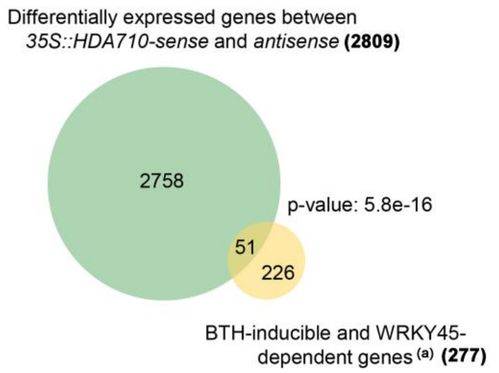

Locus ID	*35S::HDA710-sense /antisense*			*WRKY45* -kd #3 (24hr) [(a)]			Description
	Fold change	Changed	FDR	BTH response in UT (BTH/mock fold change)	WRKY45 dependence (BTH-treated UT/BTH-treated *WRKY45* -kd fold change)	FDR (BTH-responsive and WRKY45-dependent)	
LOC_Os09g25070	2.27	up	3.15E-04	227.39	2.48	1.20E-01	OsWRKY62
LOC_Os02g12380	488.49	up	3.15E-04	4.49	2.89	4.54E-02	HDA710
LOC_Os10g22310	2.26	up	3.15E-04	15.92	4.71	1.70E-02	OsGSTU35
LOC_Os10g38360	1.94	up	3.15E-04	12.51	4.70	7.80E-02	OsGSTU12
LOC_Os06g11240	1.86	up	3.15E-04	11.02	3.34	2.84E-02	OsOPR4
LOC_Os02g51930	2.04	up	2.54E-02	8.02	15.65	1.16E-01	UDP-glucuronosyl
LOC_Os08g02030	11.73	up	3.15E-04	7.75	18.76	1.48E-02	transferase family protein
LOC_Os08g26850	3.06	up	3.15E-04	7.75	2.95	1.40E-02	unknown
LOC_Os08g26840	1.97	up	3.15E-04	7.46	2.21	1.09E-01	unknown
LOC_Os03g57200	1.91	up	1.11E-02	6.23	5.03	1.09E-01	OsGSTU1
LOC_Os03g08320	2.03	up	3.15E-04	5.67	2.07	1.41E-01	OsJAZ11
LOC_Os11g30760	2.72	up	3.15E-04	5.33	2.09	1.38E-01	expressed protein
LOC_Os07g06850	3.56	up	3.15E-04	5.11	2.27	4.70E-02	GID1L2
LOC_Os04g51160	2.06	up	3.15E-04	4.95	3.26	1.80E-01	AOX1b
LOC_Os02g08440	1.88	up	3.15E-04	4.46	2.24	1.45E-01	OsWRKY71
LOC_Os01g60020	2.03	up	3.15E-04	3.93	2.10	9.27E-02	OsNAC4
LOC_Os02g04130	1.96	up	1.25E-02	3.72	2.10	5.70E-02	OsSGL
LOC_Os09g17560	2.83	up	3.15E-04	3.68	2.85	1.55E-01	ZRP4
LOC_Os04g30240	1.95	up	3.15E-04	3.66	4.29	8.26E-02	OsWAK60
LOC_Os04g32480	2.82	up	2.56E-02	3.27	2.72	7.62E-02	OsJAZ5
LOC_Os11g36000	2.04	up	1.58E-03	2.99	2.61	1.22E-01	receptor-like protein kinase 2 precursor
LOC_Os06g35060	3.42	up	3.15E-04	2.89	2.10	6.55E-02	heavy metal-associated protein
LOC_Os10g38740	1.71	up	5.94E-04	2.82	2.11	5.06E-02	OsGSTU50
LOC_Os01g15320	2.64	up	3.99E-02	0.46	0.40	1.66E-02	RALFL9
LOC_Os01g18170	4.21	up	3.15E-04	0.39	0.05	1.66E-01	Cupin domain containing protein
LOC_Os05g45200	2.89	up	3.15E-04	0.34	0.47	6.47E-02	UDP-glucuronosyl
LOC_Os08g32960	1.78	up	1.30E-02	0.32	0.33	1.01E-01	endonuclease/exonuclease/phosphatase family protein
LOC_Os06g36040	2.18	up	3.15E-04	0.13	0.50	2.74E-02	expressed protein
LOC_Os04g37490	0.53	down	3.15E-04	41.67	5.05	4.53E-02	SRP54
LOC_Os02g07170	0.31	down	3.50E-02	13.85	8.63	5.38E-02	MYB family transcription factor
LOC_Os01g43740	0.57	down	3.15E-04	9.88	16.03	7.57E-02	cytochrome P450 72A1
LOC_Os05g30350	0.57	down	3.15E-04	9.67	5.50	1.51E-02	Os5bglu22
LOC_Os06g11290	0.58	down	3.15E-04	5.72	4.01	3.38E-02	OsOPR1
LOC_Os03g55050	0.46	down	5.63E-03	5.08	3.31	5.23E-02	UDP-glucoronosyl
LOC_Os05g10840	0.49	down	8.57E-04	4.96	2.02	1.53E-01	calmodulin-binding protein
LOC_Os05g43390	0.47	down	3.15E-04	4.90	2.66	1.62E-01	SRP54
LOC_Os02g56370	0.54	down	8.97E-03	4.62	2.45	1.76E-01	OsWAK20
LOC_Os08g34790	0.45	down	3.15E-04	4.06	2.80	1.13E-01	Os4CL5
LOC_Os12g24650	0.20	down	6.18E-03	3.92	3.43	3.75E-02	chloroplast precursor
LOC_Os01g49720	0.53	down	4.27E-02	3.45	4.30	5.20E-02	OsGSTU39
LOC_Os01g36294	0.41	down	3.15E-04	3.45	2.16	1.85E-01	cytochrome P450
LOC_Os08g35600	0.47	down	1.16E-02	3.21	2.34	6.95E-02	tyrosine protein kinase protein
LOC_Os10g38590	0.49	down	3.15E-04	3.21	2.56	1.40E-01	OsGSTU9
LOC_Os03g12890	0.49	down	3.15E-04	3.12	2.08	1.24E-01	aminotransferase protein
LOC_Os04g05650	0.29	down	3.15E-04	3.00	2.56	1.38E-01	expressed protein
LOC_Os01g49710	0.28	down	1.58E-03	2.93	4.14	8.29E-02	OsGSTU40
LOC_Os11g44680	0.44	down	3.15E-04	2.90	3.75	5.12E-02	calmodulin binding protein
LOC_Os12g07310	0.51	down	8.11E-03	2.48	2.43	7.07E-02	citrate-binding protein precursor
LOC_Os01g49820	0.54	down	3.15E-04	2.40	2.02	3.19E-02	lipid phosphatase protein
LOC_Os01g03690	0.54	down	3.15E-04	2.36	2.22	4.95E-02	OsRLCK19
LOC_Os02g58214	0.44	down	2.15E-02	0.50	0.44	7.91E-03	expressed protein

(a) The data is from Nakayama et al. BMC Plant Biology 2013, 13:150 (Additional file 1: BTH-inducible and WRKY45-dependent genes).

Figure S6. Significance analysis of the 2809 differentially expressed genes with 277 BTH-inducible and WRKY45-dependent genes, and the detail information of the 51 overlapping genes.

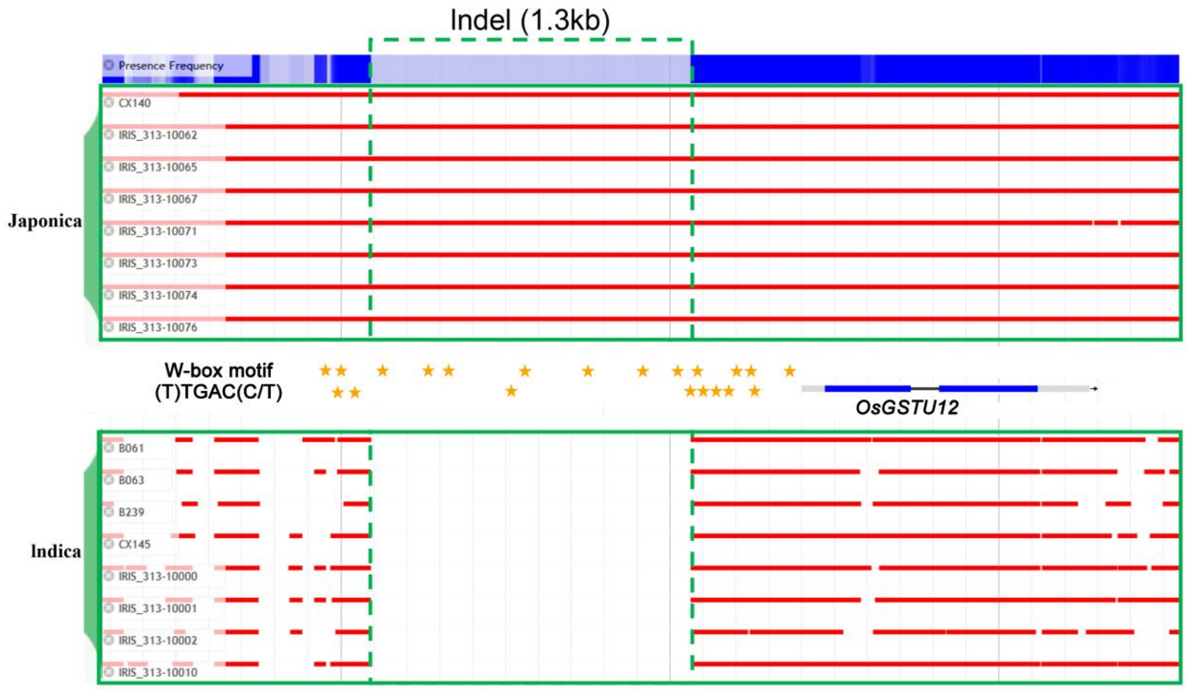

Figure S7. Natural variation of *OsGSTU12* between *japonica* and *indica* accessions.

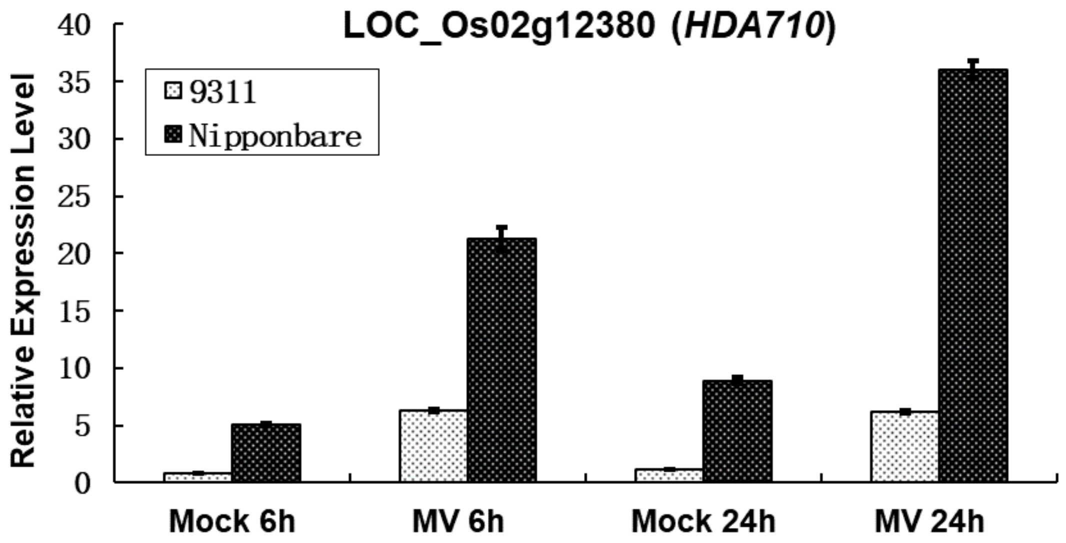

Figure S8. qRT-PCR of *HDA710* under normal conditions and MV treatment for 6 h and 24 h.

Table S1. The InDels and geographical distribution of 3,010 rice accessions (listed in separated files).

Table S2. Expression levels of 18 HDACs in Nipponbare and 9311 under control and MV treatment (listed in separated files).

Table S3. One-way ANOVA results for comparisons in Figure 2.

Comparison		df	SS	MS	F	P-value
**Figure 2E: chlorophyll content for different lines**	Genotype	4	1.157303	0.289326	79.80958	**7.30E-14**
	error	25	0.09063	0.003625		
	Total	29	1.247933			
**Figure 2F: relative electric conductivity for different lines**	Genotype	4	464.4593	116.1148	5.698979	**5.40E-03**
	error	15	305.6201	20.37		
	Total	19	770.0794			

df: degree of freedom; SS: stdev square; MS: mean square

Table S4. RNA-seq mapping rates for Nipponbare, *35S:HDA710-sense*, and *35S:HDA710-antisense* lines.

Sample	Reads all	Mapped reads	concordant pair alignment rate
Nipponbare rep1	23835875	21756156	91.3%
Nipponbare rep2	25893942	23702511	91.5%
Nipponbare rep3	27666295	25080401	90.7%
35S::HDA710-sense rep1	26722652	23979051	89.7%
35S::HDA710-sense rep2	21056503	18694249	88.8%
35S::HDA710-sense rep3	23018568	20773662	90.2%
35S::HDA710-antisense rep1	35292981	31878942	90.3%
35S::HDA710-antisense rep2	19678327	17973886	91.3%
35S::HDA710-antisense rep3	20581258	18739364	91.1%

Table S5. RNA-Seq of *HDA710*-antisense, *HDA710*-sense, and Nipponbare lines (listed in separated files).

Table S6. Top 300 co-expressed genes of *HDA710* (listed in separated files).

Table S7. Primers for qRT-PCR and vector construction.

**Primer ID**	**Forward**	**Reverse**
HDA710-sense (for qRT-PCR)	AGTACCAGAACCGATGGCAG	GTTGATGGCCATCGAATTTG
HDA710-antisense (for qRT-PCR)	AGGCAATACTGGACGTGGAG	TTCGCCTGAAAGTTCCATCT
GSTU12 (for qRT-PCR)	ATGAGGGCGTCAAGAGGTT	AGCTCGATCAGCTTGTCCAC
HDA710-sense (for vector)	GCTCTAGAATGGACCCCTCGTCGGC	GGGGTACCCTCATACATAAAAACGTAGGAA
HDA710-antisense (for vector)	GGGGTACCATGGACCCCTCGTCG	GCTCTAGACTCATACATAAAAACGTAGGAA
OsGSTU12 (for vector)	TCCCCCGGGGGACAGAGCTCGCAACTCACAAG	CGGGGTACCCCGAGGGCATACATGTGCTTCTT
